# Toll-like receptor 4 inhibition within the paraventricular nucleus attenuates blood pressure and inflammatory response in a genetic model of hypertension

**DOI:** 10.1186/s12974-015-0242-7

**Published:** 2015-02-18

**Authors:** Rahul B Dange, Deepmala Agarwal, Ryoichi Teruyama, Joseph Francis

**Affiliations:** Comparative Biomedical Sciences, School of Veterinary Medicine, Louisiana State University, 1909 Skip Bertman Drive, Baton Rouge, LA 70803 USA; William Hansel Cancer Prevention Laboratory, Pennington Biomedical Research Center, 6400 Perkins Road, Baton Rouge, LA 70808 USA; Department of Biological Sciences, College of Science, Louisiana State University, 202 Life Sciences Building, Baton Rouge, LA 70803 USA

**Keywords:** Paraventricular nucleus, Hypertension, TLR4, Cytokines, IL-10, NFκB

## Abstract

**Background:**

Despite the availability of several antihypertensive medications, the morbidity and mortality caused by hypertension is on the rise, suggesting the need for investigation of novel signaling pathways involved in its pathogenesis. Recent evidence suggests the role of toll-like receptor (TLR) 4 in various inflammatory diseases, including hypertension. The role of the brain in the initiation and progression of all forms of hypertension is well established, but the role of brain TLR4 in progression of hypertension has never been explored. Therefore, we investigated the role of TLR4 within the paraventricular nucleus (PVN; an important cardioregulatory center in the brain) in an animal model of human essential hypertension. We hypothesized that a TLR4 blockade within the PVN causes a reduction in mean arterial blood pressure (MAP), inflammatory cytokines and sympathetic drive in hypertensive animals.

**Methods:**

Spontaneously hypertensive rats (SHR) and normotensive Wistar Kyoto (WKY) rats were administered either a specific TLR4 blocker, viral inhibitory peptide (VIPER), or control peptide in their PVN for 14 days. MAP was recorded continuously by radiotelemetry. PVN and blood were collected for the measurement of pro-inflammatory cytokines (Tumor Necrosis Factor (TNF)-α, interleukin (IL)-1β), anti-inflammatory cytokine IL-10, inducible nitric oxide synthase (iNOS), TLR4, nuclear factor (NF) κB activity and plasma norepinephrine (NE) and high mobility group box (HMGB)1 expression, respectively.

**Results:**

Hypertensive rats exhibited significantly higher levels of TLR4 in the PVN. TLR4 inhibition within the PVN attenuated MAP, improved cardiac hypertrophy, reduced TNF-α, IL-1β, iNOS levels, and NFκB activity in SHR but not in WKY rats. These results were associated with a reduction in plasma NE and HMGB1 levels and an increase in IL-10 levels in SHR.

**Conclusions:**

This study demonstrates that TLR4 upregulation in PVN plays an important role in hypertensive response. Our results provide mechanistic evidence that hypertensive response in SHR are mediated, at least in part, by TLR4 in the PVN and that inhibition of TLR4 within the PVN attenuates blood pressure and improves inflammation, possibly via reduction in sympathetic activity.

## Background

According to current statistics, hypertension affects more than 33% of US adults [1] and it has emerged as one of the most common chronic disease in the United States. Hypertension is characterized by elevated blood pressure, cardiac hypertrophy and chronic low-grade inflammation. Several mechanisms that have been shown to exist in hypertension include excessive vasoconstriction, commonly involving the endogenous peptides, angiotensin II and endothelin, or deficient vasodilatation, often involving nitric oxide (NO), dysregulation of adrenergic mechanisms [2], increase in the total peripheral resistance either due to increase in the contractile responses or a decrease in relaxant responses in resistance arteries [3]. Nevertheless, uncontrolled hypertension may lead to various end organ injuries including, but not limited to, cardiac remodeling [4-6], renal dysfunction [5,6] , retinopathy [7] coronary heart disease, heart failure and stroke. A critical player leading all these events is the immune system, which does so by participating in an inflammatory cascade in the cardiovascular, renal and central nervous system (CNS) [8-10]. In the CNS, the brain is the central organ playing an important in fluid homeostasis and sympathetic regulation of blood pressure (BP). Unlike previous studies indicating the role played by the brain in late stage hypertensive disease, a growing body of evidence suggests the role of the brain in the initiation and progression of all forms of hypertension including essential hypertension [11].

Innate immunity is the first line of defense against pathogens, which is initiated by infection or tissue insult. This critical response is initiated by identification of pathogen-associated molecular patterns by pathogen-recognition receptors present on cell membrane [12]. One such pathogen-recognition receptor includes toll-like receptors (TLRs), which are major components of the innate immune system. TLR is an evolutionarily conserved receptor family that was initially discovered to be responsible for determining the dorsal-ventral axis in *Drosophila*. Later, TLRs were found controlling host defense against microbes in plants, vertebrates, and mammals. A total of 13 isoforms of TLRs (TLR1, 2, 3, *etcetera*) have been identified in mammalian cells, including leukocytes, cardiomyocytes and endothelial cells [13,14], each of which recognizes specific pathogen-associated molecular patterns (PAMPs) or damage-associated molecular patterns (DAMPs). Of several DAMPs, high mobility group box-1 (HMGB1) is the most important DAMP that has been implicated in various inflammatory conditions. In response to injury or infection, HMGB1 is released actively by immune cells and passively by insulted cells into the extracellular space and initiates an inflammatory response [15-17].

Hypothalamic paraventricular nucleus (PVN) is the most important cardiovascular regulatory center within the brain, playing a major role in sympathetic control of BP. The PVN regulates sympathetic output, baroreflex function and salt appetite [4,18], thus acting as an important endocrine-autonomic control area in the brain. Evidence suggests that the increased pro-inflammatory cytokines(PICs) within the PVN of the brain play an important role in the development of hypertension [19,20]. It has been observed that, administration of PICs into the PVN causes increased renal sympathetic output and mean arterial pressure [21]. A cytokine-induced increase in inducible nitric oxide synthase (iNOS) has also been found to assist in the development of hypertension [22,23]. It is now well established that an increase in PICs causes increased production of reactive oxygen species (ROS), leading to increased activity of downstream transcription factor, nuclear factor-kappa B (NFκB), which in turn further upregulates PICs. This vicious cycle ultimately contributes to an altered pathophysiologic phenotype and high blood pressure.

A growing body of evidence suggests the innate immune system plays a role in several inflammatory processes affecting the cardiovascular system, including hypertension. Although TLR is a critical component of the innate immune system, the role of TLR in the etiology of hypertension is not well understood. Based on previous reports from our laboratory and others, out of 13 TLRs, TLR4 can be implicated in the pathogenesis of hypertension. Given the role of PVN in initiation of high blood pressure, it is imperative to investigate the role of TLR4 within the PVN in modulating inflammatory response in hypertension. In addition, the underlying molecular mechanisms in TLR4 mediated inflammatory response in hypertension have never been studied. Therefore, we hypothesize that TLR4 signaling in the PVN of the brain might be one of the main factors for the establishment of hypertension and cardiac remodeling observed in the SHR rats. In this study, we aim to investigate the role of TLR4 in the PVN of SHR rats, a model of human essential hypertension, and evaluate if the TLR4 blockade within the PVN has any protective role in hypertension, cardiac function, inflammatory cytokines in the brain and plasma, and sympathetic activity. The outcomes of this study will help us to understand pathogenesis of hypertension and develop newer therapeutics for its effective control.

## Methods

### Ethics statement

All of the animal procedures in this study were reviewed and approved by the Louisiana State University Institutional Animal Care and Use Committee (IACUC) in accordance with the National Institutes of Health Guide for the Care and Use of Laboratory Animals.

### Experimental design

Male spontaneously hypertensive (SHR) rats and their normotensive control Wistar Kyoto (WKY) rats (10 to 12 weeks old, 230 to 340 g) were used in this study. They were housed in temperature-controlled (23 ± 2°C) and light-controlled (lights on between 7 AM and 7 PM) animal quarters and were provided with chow *ad libitum*. The rats were surgically implanted with radiotelemetry transmitters for continuous monitoring of mean arterial pressure (MAP). Bilateral canulae were implanted into the PVN of these animals for infusion of viral inhibitory peptide of TLR4 (VIPER, a specific TLR4 receptor blocker; *Imagenex Corp, San Diego, CA, USA*) or control peptide (CP) (40 μg/kg/day; *Imagenex Corp, San Diego, CA, USA*) dissolved in aCSF (artificial CSF) through osmotic minipump (14 days, infusion rate 0.11 μl/h; Alzet, model 1004) for two weeks. VIPER specifically inhibit TLR4 signaling by binding its adaptor proteins Mal/TIRAP and TRAM [24,25].The dose of the VIPER used was assessed from a pilot study in rats where three different doses of 10, 40 and 160 μg/kg/day were used. The 40 μg/kg/day was found to be optimal, whereas the highest dose caused mortality and lowest dose did not produce complete inhibition of TLR4 receptors as measured by immunofluorescence staining and western blot results (data not shown). The rats were divided into four groups (n =20/group): (1) WKY + CP; (2) WKY + VIPER; (3) SHR + CP; (4) SHR + VIPER. At the end of the study, rats were euthanized using CO_2_ inhalation; the brains and heart tissues (left ventricle) were collected, and immediately frozen on dry ice until further analysis.

### Blood pressure measurements

MAP was recorded continuously in conscious rats (n = 14/group) using surgically implanted radio-telemetry transmitters (Model TA11PA-C40, Data Sciences International, St. Paul, MN, USA), as described previously [26,27]. The rats were anesthetized with a ketamine (90 mg/kg) and xylazine (10 mg/kg) mixture (i.p.) and placed dorsally on a heated surgical table. Limb withdrawal response to toe pinching was used to ascertain the adequacy of anesthesia. Through a small incision on the ventral surface of left leg, femoral artery and vein were exposed and dissected apart. The femoral artery was ligated distally, and a small clamp was applied to temporarily stop the blood flow. The catheter tip was introduced through a small incision in the femoral artery, advanced into the abdominal aorta such that the catheter tip was distal to the renal arteries, and secured in place. The transmitter body was placed in to the abdominal cavity and secured to the abdominal wall. Following implantation, abdominal musculature and skin layers were sutured and closed. After surgery, all rats received benzathine penicillin (30,000 U, i.m.) and buprenorphine (0.1 mg/kg, s.c.), which was repeated 12 h postoperatively. The rats were allowed to recover for 7 days from surgical trauma.

### Intra-paraventricular nucleus cannula implantation

The rats were implanted with intra-PVN cannula for infusion of VIPER or CP, as described previously [19]. Briefly, the rats were anesthetized with a ketamine (90 mg/kg) and xylazine (10 mg/kg) mixture (i.p.), and then placed in a stereotaxic instrument (Kopf instruments; Tujunga, CA). The adequacy of anesthesia was monitored by limb withdrawal response to toe pinching. Bregma was identified and according to Paxinos and Watson [28] rat atlas, coordinates for the PVN were obtained at 1.8 mm posterior and 7.9 mm ventral to the zero level. A custom-made bilateral cannulae (Plastic One; Roanoke, VA, USA) was implanted in to the PVN of animals, and fixed to the cranium using small screws and dental cement. A 14-day miniosmotic pump was implanted subcutaneously and connected to the infusion cannula through a sterile vinyl tubing to deliver VIPER or CP in the PVN of the brain. Cannula placement within the PVN was examined during cryostat slicing with crystal violet staining prior to PVN punching and immunofluorescence sectioning. Rats that had malfunctioning pumps (such as tube detachment from pump or cannula, based on postmortem analysis) or received unilateral treatment into the PVN were removed from the final analysis (success rate: bilateral cannulation, 80%; n = 25).

### Paraventricular nucleus collection, RNA isolation, and real-time RT-PCR

Freshly frozen brains were cut on a cryostat to get coronal brain sections, which were mounted on slides for punch microdissection. The PVN punches were made from the frozen brain sections using Stoelting brain punch (Stoelting, IL, USA) as described earlier [29,30]. Semi-quantitative real-time RT-PCR was used to determine the mRNA levels of TNF-α, IL-1β, IL-10, iNOS, and TLR4 in the PVN tissues (n = 8/group) and ANP (Atrial Natriuretic Peptide- a marker of cardiac hypertrophy) in left ventricular tissues by using specific primers (Table 1). Total RNA isolation, cDNA synthesis and RT-PCR were performed as previously described [31]. DNAase- treatment was performed to prevent cDNA contamination from genomic DNA. Semi-log amplification curves were evaluated by the comparative quantification method (2^-ΔΔCT^), and GAPDH was used for normalization of all reported gene expression levels. The data are presented as the fold change of the gene of interest relative to that of the control group.

**Table 1:** **Rat primers used for real-time RT-PCR**

**Gene**	**Sense**	**Antisense**
GAPDH	Agacagccgcatcttcttgt	cttgccgtgggtagagtcat
TNF-α	gtcgtagcaaaccaccaagc	tgtgggtgaggagcacatag
IL-1β	gcaatggtcgggacatagtt	agacctgacttggcagaga
IL-10	gggaagcaactgaaacttcg	atcatggaaggagcaacctg
iNOS	ccttgttcagctacgccttc	ggtatgcccgagttctttca
TLR4	ggctgtggagacaaaaatgacctc	aggcttgggcttgaatggagtc

IL, Interleukin; TNF-α, Tumor necrosis factor-alpha; iNOS, Inducible nitric oxide synthase; GAPDH, Glyceraldehyde 3-phosphate dehydrogenase; TLR4, Toll-like receptor 4.

### Western blot analysis

The tissue homogenate from the PVN was subjected to western blot analysis (n = 6/group) for determination of protein levels of PICs (TNF-α, IL-1β), IL-10, iNOS, TLR4 and GAPDH. The western blot was performed by standard protocol, as described previously [32]. Briefly, the heart tissues were homogenized in 500 ul (100 μl for PVN tissue) of RIPA (Radioimmunoprecipitation Assay Buffer) lysis buffer (Cell Signaling Technology, Inc., MA, USA) containing protease inhibitor cocktail. The protein was extracted from the homogenates, and the protein concentration in the lysate was measured using a Bradford assay using BSA standards. Protein extracts (40 μg) were combined with an equal volume of 2X Laemmli loading buffer, boiled for 5 minutes and electrophoresed on 10 to 15% SDS-polyacrylamide gels. The proteins were then electroblotted onto polyvinylidene fluoride membranes (Immobilon-P, Millipore, MA, USA). Nonspecific binding was blocked by incubating the membranes in 1% casein in phosphate-buffered saline-Tween for 1 h at room temperature (RT). Blots were then incubated overnight at 4°C with the primary antibodies. Specific antibodies used included TNF-α, IL-1β, iNOS, GAPDH, and TLR4, at 1:1,000 dilution; IL-10, at 1:500 dilution. The following antibodies were commercially obtained: TNF-α, and TLR4 (mouse monoclonal; Abcam Inc, MA; ab22048); IL-1β, iNOS, and GAPDH (Santa Cruz Biotechnology, Santa Cruz, CA, USA); and IL-10 (Abbiotec, LLC, San Diego, CA, USA). After washing with wash buffer (1X TBS, 0.1% Tween-20) four times for 10 min each time at RT, blots were then incubated for 1 h with secondary antibody (1:10,000 dilution, Santa Cruz Biotechnology, CA, USA) labeled with horseradish peroxidase. Immunoreactive bands were visualized using enhanced chemiluminescence (ECL Plus, Amersham, GE Healthcare Bio-Sciences, PA, USA), band intensities were quantified using a VersaDoc MP 5000 imaging system (Bio-Rad, CA, USA), and bands were normalized with GAPDH.

### Immunofluorescence double labeling

Immunofluorescence procedures were carried out as previously described [33]. Coronal brain sections were cut with a freezing microtome (Leica, NJ, USA) at 25 μm at the mid-rostrocaudal levels of the PVN as described earlier [34]. Briefly, sections were preincubated with 1% H_2_O_2_ in 0.1 M PBS (30 min), blocked with 2% normal goat serum (KPL, CA, USA) (1 hr at room temperature), and then incubated (overnight at 4°C) with the primary antibodies in primary antibody diluents (containing, normal goat serum 10% Triton X, in 0.1 M PBS). Specific primary antibodies used included mouse anti-TLR4 (1:500) (Abcam, USA; mouse monoclonal, ab22048), rabbit anti-TLR4 (1:1000; ab13556), rabbit anti-Neuronal Nuclei (NeuN) (1:500) (Abcam, MA,USA), rabbit anti-glial fibrillary acid protein (GFAP) (1:100) (Abcam, MA, USA), or mouse anticluster of differentiation 11b (CD11b) (1:500) (Abcam, MA, USA). Conjugated secondary antibodies were used to detect the primary antibody, which included Alexa Fluor 594 goat anti-mouse IgG (H + L) (1:1000), Alexa Fluor 488 goat anti-rabbit IgG (H + L) (1:1000) (Cell Signaling Technology, MA, USA), or fluorescein isothiocyanate-goat anti-mouse IgG antibodies (1:200) (Zymed, CA, USA) in 0.1 M PBS (1 hr at RT), and then sections were mounted in ProLong Gold antifade reagent (P36930, Invitrogen, NY,USA). Immunoreactivity was visualized using the BX53 microscope (Olympus America Inc, PA, USA) and digitized at 20x and 40x using DP2-BSWVer2.2software (https://support.olympus.co.jp/cf_secure/en/lisg/bio/download/ga/dp2bsw0202/). Negative control sections were incubated with only PBS and no positive staining was observed. As an additional control, only the secondary antibody (in absence of the primary) was used for TLR4, which prevented TLR4 staining (data not shown).

### Determination of NFκB activity in the paraventricular nucleus

The binding activity of free NFκB/p65 in nuclear extracts of PVN tissue (n = 6/group) was assessed using an ELISA kit (Active Motif, CA, USA). The method of extraction and data analysis was as per the manufacturer’s guidelines and as described previously [19]. This procedure yields 0.15 to 0.25 mg of PVN tissue nuclear extract at 3 to 5 mg/ml. Active ELISA detection levels for NFκB/p65 from nuclear extracts were 0.5 to 1 μg per well at 450 nm.

### Detection of plasma norepinephrine and HMGB1

Norepinephrine (NE) and HMGB1 were measured in plasma samples (n =8/group) using a norepinephrine ELISA kit (Abnova, Taiwan) and a HMGB1 ELISA kit (MyBioSource, CA, USA), respectively as per the manufacturer’s guidelines.

### Statistical analysis

All results are expressed as mean ± SEM. Statistical analysis was done by either a two-way ANOVA or a one-way ANOVA with a Bonferroni post hoc test using Graph Pad Prism software (version 5.0; GraphPad Software, San Diego California, USA). MAP data were analyzed by repeated-measures ANOVA to examine with-in group changes over time. A value of *P* <0.05 was considered statistically significant.

## Results

### Toll-like receptor 4 is highly expressed in the neurons and microglia of paraventricular nucleus in hypertensive rats

**Figure 1:**
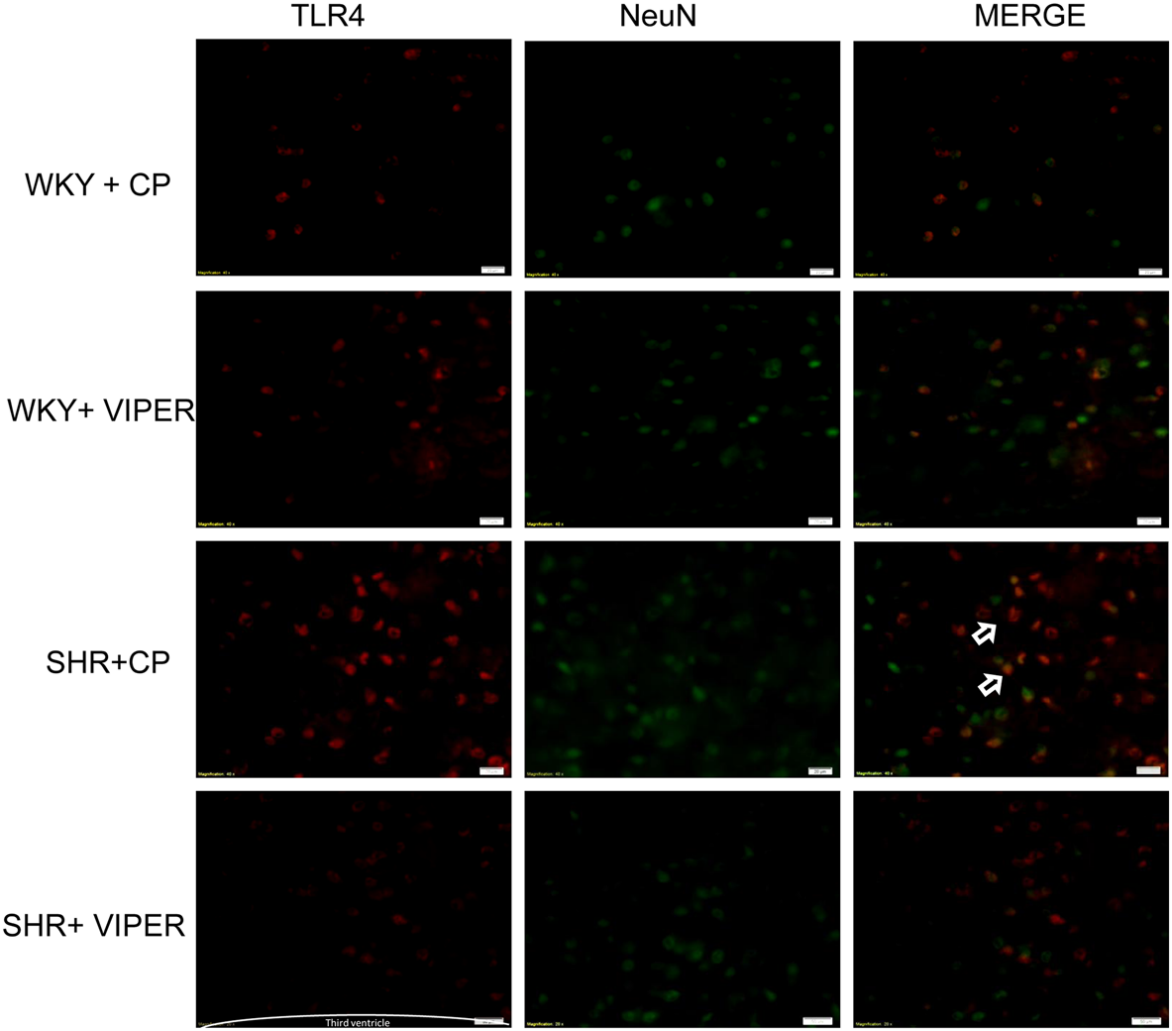
**An immunofluorescence double labeling image (x 20) showing the effects of intra-PVN infusion of VIPER on protein expression of TLR4 and NeuN in the PVN of WKY and SHR rats. n = 5/group.** SHR + CP rats showed higher levels of immunofluorescence for TLR4 within the neurons of PVN, whereas, VIPER infusion in these rats caused significant reduction in TLR4 expression. Arrow indicates double- labeled cells.VIPER infusion in saline-infused rats did not have any effects. Scale bar 20 μm: CP, control peptide; NeuN, neuronal nuclei; PVN, paraventricular nucleus; SHR, spontaneously hypertensive rat; TLR4, Toll-like receptor 4; VIPER, viral inhibitory peptide of TLR4; WKY, wistar-Kyoto.

**Figure 2:**
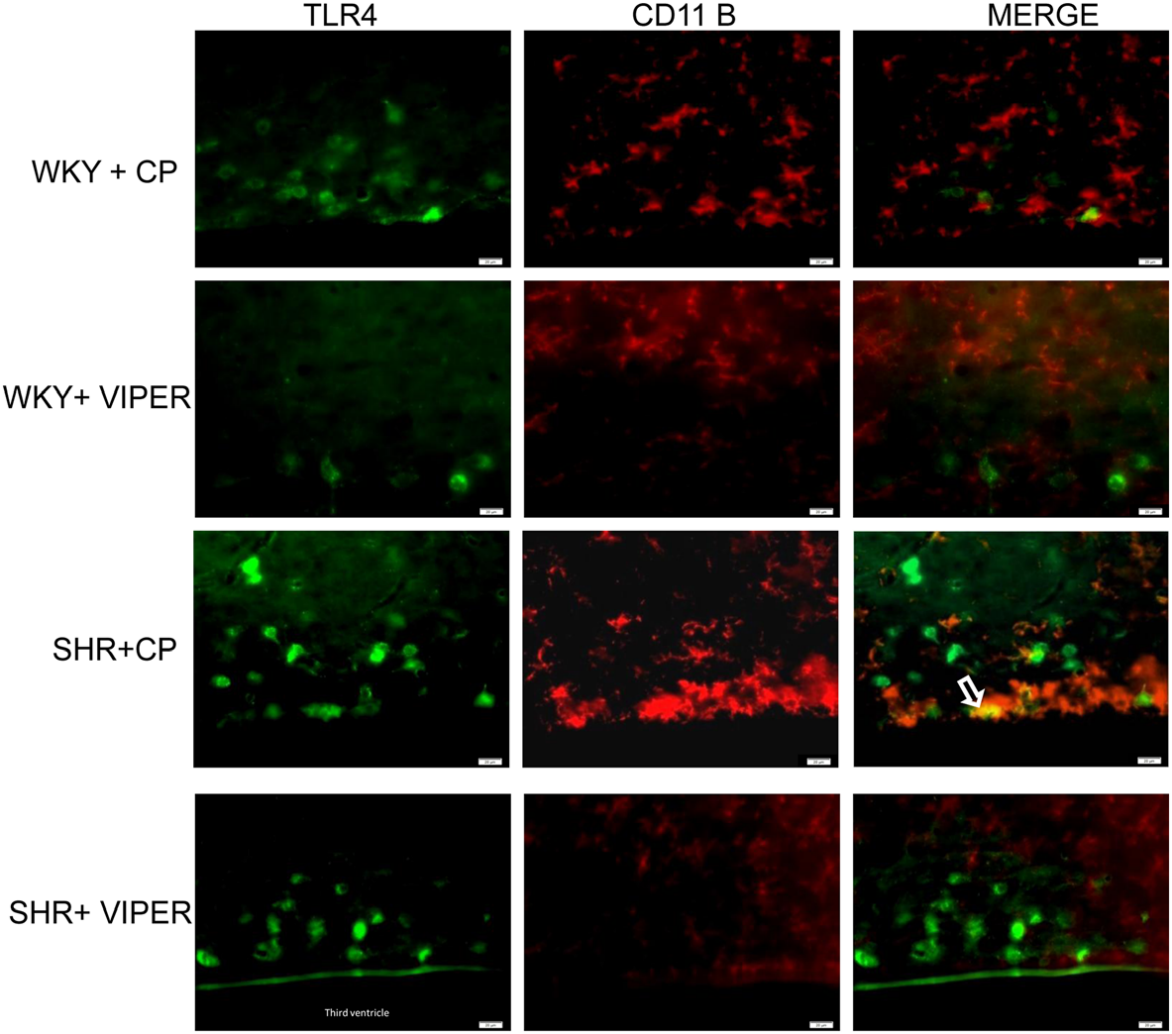
**An immunofluorescence double labeling image (x 40) showing the effects of intra-PVN infusion of VIPER on protein expression of TLR4 and CD11B in the PVN of WKY and SHR rats. SHR + CP rats showed modest expression of TLR4 within the microglia of PVN, whereas, VIPER infusion in these rats caused significant reduction in TLR4 expression.** Arrow indicates double-labeled cells.VIPER infusion in saline-infused rats did not have any effects. n = 5/group. Scale bar 20 μm : *CD11B,* cluster of differentiation molecule 11B; CP, control peptide; PVN, paraventricular nucleus; SHR, spontaneously hypertensive rat; TLR4, Toll-like receptor 4; VIPER, viral inhibitory peptide of TLR4; WKY, wistar-Kyoto.

**Figure 3:**
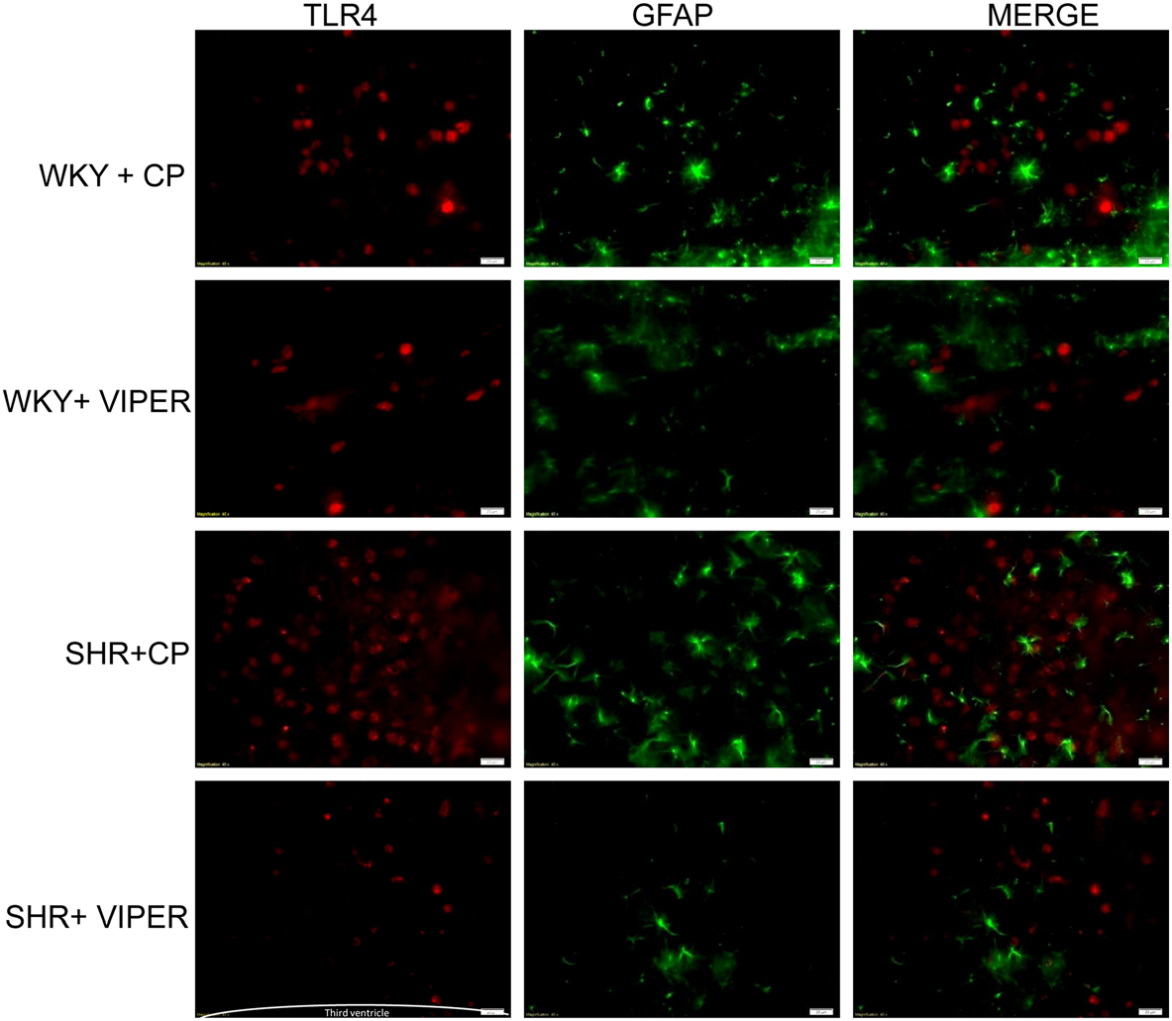
**An immunofluorescence double labeling image (x 20) showing the effects of intra-PVN infusion of VIPER on protein expression of TLR4 and GFAP in the PVN of WKY and SHR rats. n = 5/group. Scale bar 20 μm** : GFAP, glial fibrillary acidic protein; PVN, paraventricular nucleus; SHR, spontaneously hypertensive rat; TLR4, Toll-like receptor 4; VIPER, viral inhibitory peptide of TLR4; WKY, wistar-Kyoto.

**Figure 4:**
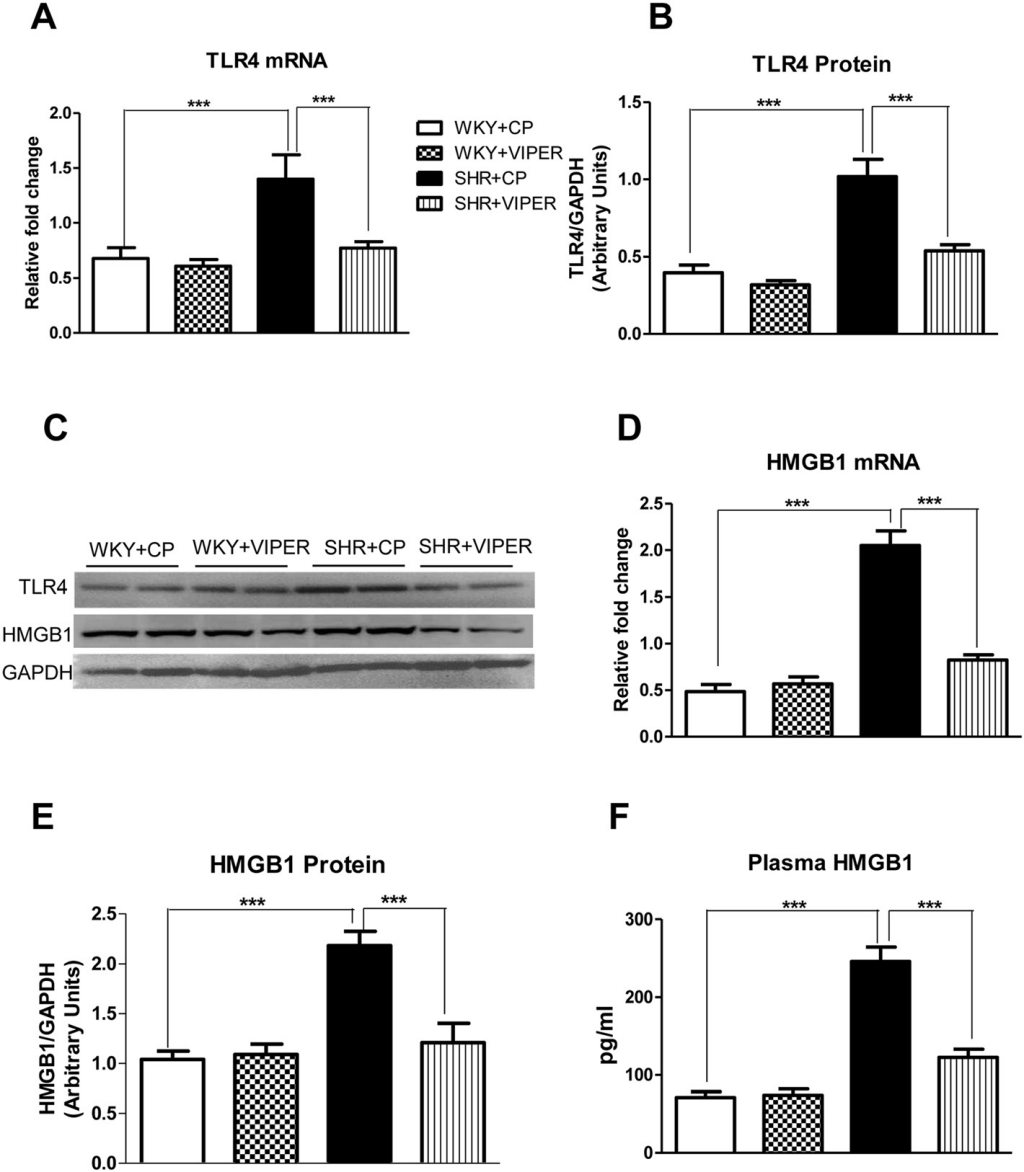
**Effects of PVN infusion of VIPER on gene and protein expression of TLR4 and HMGB1 in WKY and SHR rats. (A)** mRNA expression of TLR4; **(B)** protein expression of TLR4; **(C)** A representative immunoblot; **(D)** mRNA expression of HMGB1; **(E)** protein expression of HMGB1; and **(F)** plasma levels of HMGB1. Values are means ± SE. n = 8 in each group for mRNA analysis, n = 5 in each group for western blot, and n = 8 for plasma HMGB1. ***P <0.001 : HMGB1, high mobility group box 1; PVN, paraventricular nucleus; SHR, spontaneously hypertensive rat; TLR4, Toll-like receptor 4; VIPER, viral inhibitory peptide of TLR4; WKY, wistar-Kyoto.

Immunofluorescence staining of the PVN sections showed that TLR4 is highly expressed in SHR + CP groups when compared to WKY + CP (Figures 1, 2 and 3). Cell-type distribution of TLR4 was further investigated in the PVN of all four groups using a double-labeling immunofluorescence technique. The frozen floating sections were labeled with TLR4 antibody and one of the following: neuronal nuclei (NeuN), glial fibrillary acidic protein (GFAP) or anti-CD11b antibodies. NeuN, GFAP and anti-CD11b were used to identify neurons, astrocytes and microglia, respectively. An overwhelming majority of TLR4 (red) was co-localized with NeuN-positive neurons (green) (Figure 1) in SHR + CP rats. Some of the TLR4-positive cells (green) were also labeled with CD11b-positive microglia/macrophage cells (red) (Figure 2); whereas, almost none of the TLR4-positive cells (red) were co-localized with GFAP-positive astrocytes (green) in the PVN of SHR + CP rats (Figure 3). These results indicated that TLR4 is mainly expressed in the neurons and microglia of the PVN. Furthermore, chronic intra-PVN infusion of VIPER in SHR caused an apparent reduction in TLR4 fluorescent staining in the PVN. These results corroborated with RT-PCR and western blot analysis confirming the efficacy of VIPER in inhibiting TLR4 expression within the PVN (Figure 4A-C).

### Toll-like receptor 4 blockade reduced HMGB1 levels in circulation and in the paraventricular nucleus of hypertensive rats

SHR + CP group had increased gene and protein expression of HMGB1 when compared to WKY + CP rats (Figure 4C-E). Furthermore, circulating plasma levels of HMGB1 was significantly higher in SHR + CP in comparison with WKY + CP rats (Figure 4F). Interestingly, inhibition of TLR4 using VIPER in the PVN caused significant reduction in plasma and PVN levels of HMGB1 in hypertensive rats when compared to WKY + CP group. VIPER infusion did not affect HMGB1 in WKY rats.

### Toll-like receptor 4 blockade in the paraventricular nucleus attenuated mean arterial pressure in hypertensive rats

**Figure 5:**
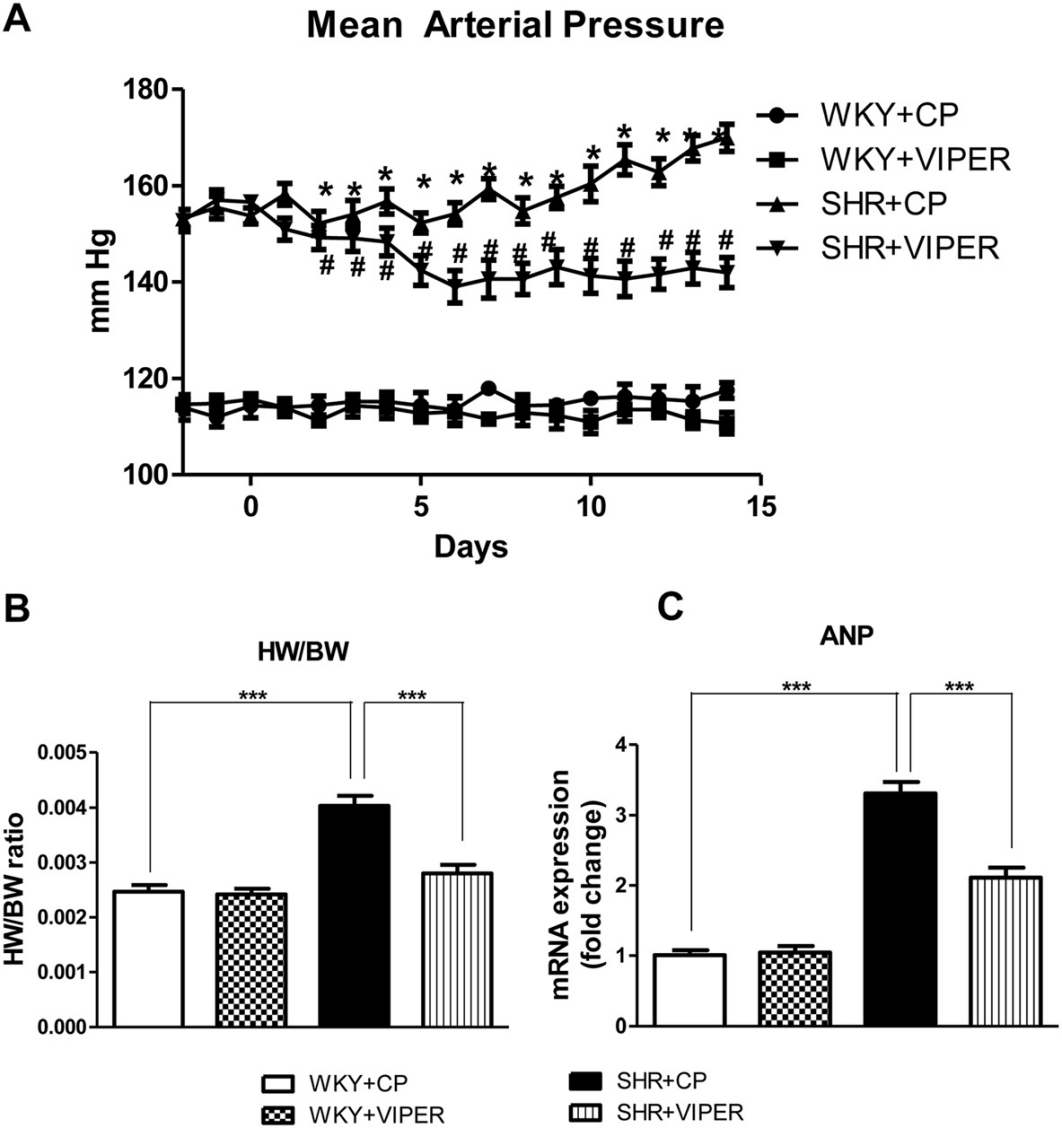
**Effect of bilateral intra-PVN infusion of VIPER on mean arterial blood pressure (MAP, in millimeters of mercury) and cardiac hypertrophy in WKY and SHR rats. (A)** SHR + CP group had significantly increased MAP when compared to WKY + CP rats. Interestingly, infusion of VIPER in PVN of SHR rats for 14 days resulted in significant decrease in MAP, starting from day 4 of VIPER infusion; **(B)** heart weight to body weight ratio; and **(C)** mRNA expression of atrial natriuretic peptide (ANP) in tissue obtained from left ventricle. Values are mean ± SE; n = 8/group for mRNA analysis and n = 14 for MAP. *P <0.05 SHR + CP versus WKY + CP; #*P* < 0.05 SHR + VIPER versus SHR + CP; ***P <0.001 : ANP, Atrial Natriuretic Peptide; CP, control peptide; MAP, mean arterial pressure; PVN, paraventricular nucleus; SHR, spontaneously hypertensive rat; TLR4, Toll-like receptor 4; VIPER, viral inhibitory peptide of TLR4; WKY, wistar-Kyoto.

To assess the effect of inhibition of TLR4 in the PVN on hypertensive response in animal model of essential hypertension, MAP was continuously monitored using implanted radio-telemetry devise. As seen in Figure 5 A, SHR + CP rats exhibited a significant increase in MAP when compared to WKY + CP rats. Whereas, SHR + VIPER rats had significantly reduced MAP starting from day 4, and it remained lower for the duration of the study (at day 14, 170 ± 1.1 versus 142 ± 1.5 mmHg, *P* <0.05) when compared to SHR + CP rats. WKY + CP and WKY + VIPER did not differ significantly.

### Toll-like receptor 4 blockade in the paraventricular nucleus attenuated cardiac hypertrophy in hypertensive rats

To assess cardiac hypertrophy in the rats, heart weight was recorded at the end of experimental period. The heart weight to body weight ratio (HW/BW) was calculated as a predictor of cardiac hypertrophy. Additionally, ANP, a molecular marker of cardiac hypertrophy was estimated in heart tissue using real time RT-PCR. Increased cardiac hypertrophy was observed in SHR + CP rats compared with WKY + CP rats, as indicated by a significantly increased HW/BW ratio and ANP levels in SHR + CP group (Figure 5B and C). VIPER treatment in SHR rats caused a significant decrease in the HW/BW ratio as well as ANP levels in comparison with SHR + CP, suggestive of reduced cardiac hypertrophy by blockade of TLR4 within the PVN of SHR. These data suggest a role for TLR4 in the PVN of the brain on MAP regulation and cardiac hypertrophy in the essential hypertension.

### Toll-life receptor 4 blockade altered expression of pro- and anti-inflammatory cytokines in hypertensive rats

**Figure 6:**
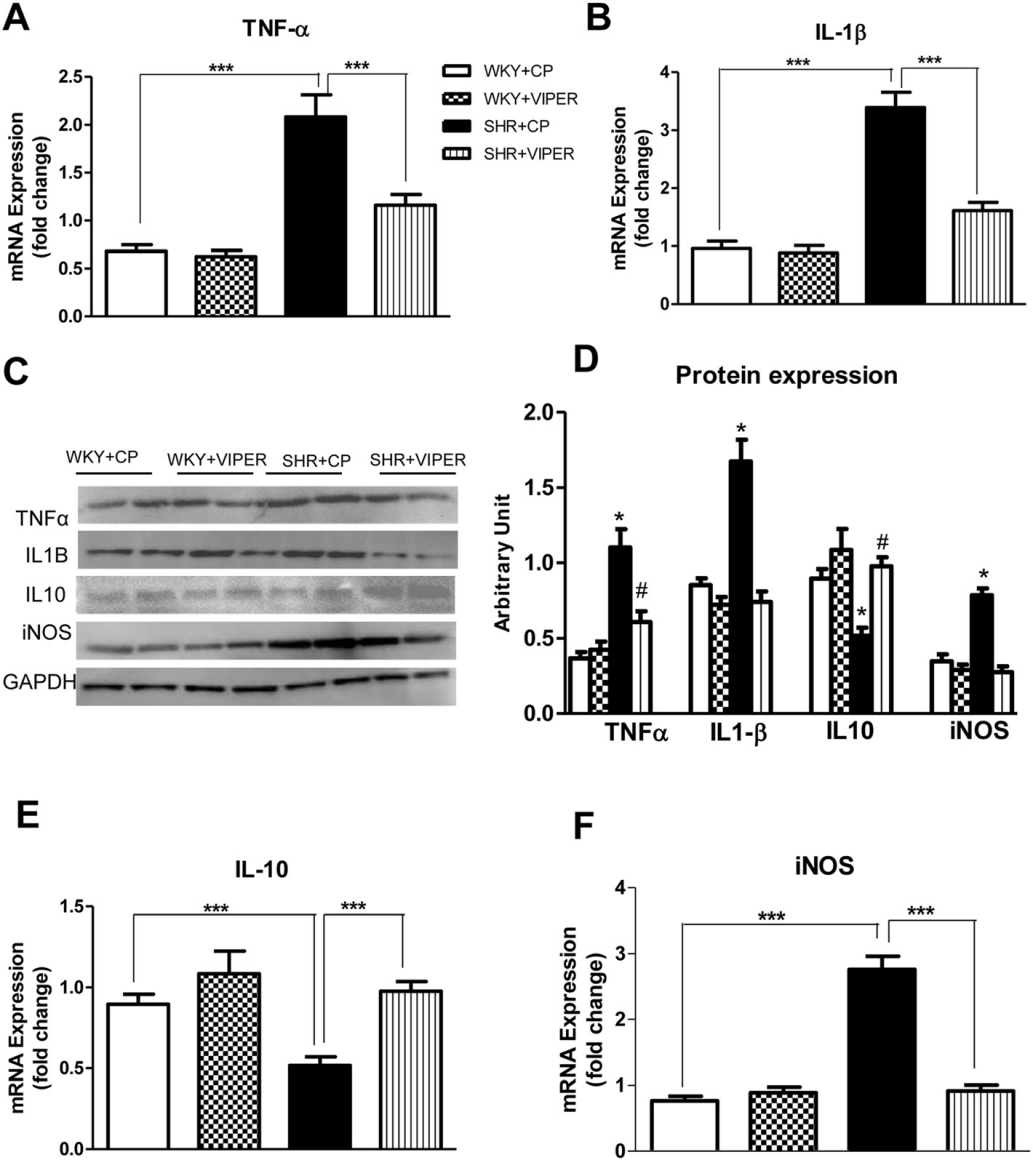
**Effects of bilateral intra-PVN infusion of VIPER on gene and protein expression of pro- and anti-inflammatory cytokines and iNOS in WKY and SHR rats. (A)** mRNA expression of TNF-α; **(B)** mRNA expression of IL1-β; **(C)** A representative immunoblot; **(D)** densitometric analysis of protein expression of TNF-α, IL1-β, IL10 and iNOS; **(E)** mRNA expression of IL10; and **(F)** mRNA expression of iNOS. Values are means ± SE. n = 8 in each group for mRNA analysis and n = 5 in each group for western blot. ***P <0.001; *P <0.05 SHR + CP versus WKY + CP; #*P* <0.05 SHR + VIPER versus SHR + CP: CP, control peptide; IL-1β, interleukin-1 beta; IL10, interleukin10; iNOS, inducible nitric oxide; PVN, paraventricular nucleus; SHR, spontaneously hypertensive rat; TLR4, Toll-like receptor 4; TNF-α, tumor necrosis factor-alpha; VIPER, viral inhibitory peptide of TLR4; WKY, wistar-Kyoto. Furthermore, IL-10 gene and protein expression in the PVN tissue was measured to assess the effect of TLR4 blockade on anti-inflammatory axis. A significant reduction in IL-10 gene and protein expression in the SHR + CP compared with the WKY + CP rats was evident (Figure 6 C and E). Interestingly, the SHR + VIPER group had significantly higher levels of IL-10 in comparison with the SHR + CP rats, whereas there was no difference between the WKY + CP and WKY + VIPER groups.

To determine the effect of TLR4 blockade within the PVN on inflammatoy response in hypertension, we examined the levels of pro-inflammatory cytokines (PICs), TNF-α and IL-1β mRNA (Figure 6A-B) and protein (Figure 6 C-D) levels in the PVN by real time RT PCR and western immunoblot, respectively. We observed that SHR + CP rats exhibited marked increase in TNF-α and IL-1β expression compared to WKY + CP rats in their PVN. Interestingly, this upregulation of TNF-α and IL-1β was significantly attenuated in SHR + VIPER group. However, VIPER infusion did not affect PICs levels in WKY rats.

Furthermore, IL-10 gene and protein expression in the PVN tissue was measured to assess the effect of TLR4 blockade on anti-inflammatory axis. A significant reduction in IL-10 gene and protein expression in the SHR + CP compared with the WKY + CP rats was evident (Figure 6 C and E). Interestingly, SHR + VIPER group had significantly higher levels of IL-10 in comparison with SHR + CP rats; whereas there was no difference between WKY + CP and WKY + VIPER groups.

### Toll-like receptor 4 blockade alters inducible nitric oxide synthase levels in the hypertensive rats

Hypertension is found to modulate iNOS expression. iNOS is induced primarily by PICs and is another marker of inflammation. We found that SHR + CP rats had marked increase in mRNA and protein levels of iNOS (Figure 6 C and F) in comparison with WKY + CP rats. Importantly, chronic infusion of VIPER in the PVN caused a significant decrease in mRNA and protein expression of iNOS in the SHR rats but not in the WKY rats.

### Toll-like receptor 4 blockade attenuates NFκB activity in hypertensive rats

**Figure 7:**
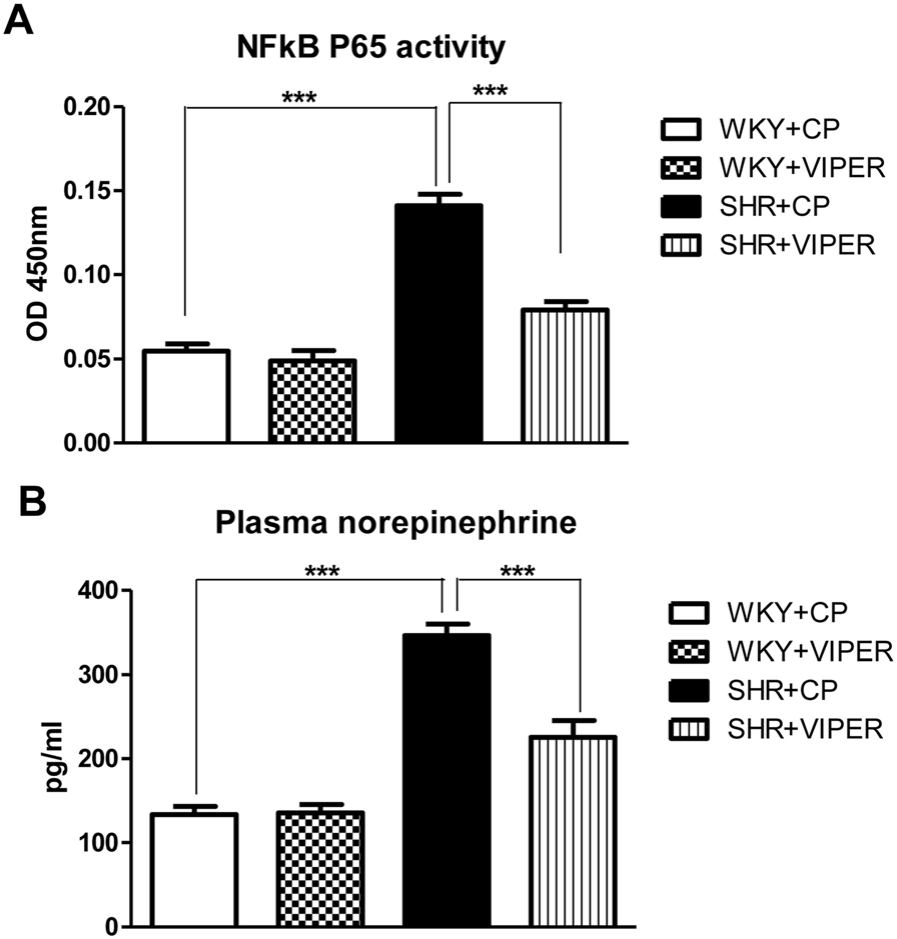
**Effects of bilateral intra-PVN infusion of VIPER on (A) NFκB activity of PVN tissue and (B) plasma levels of norepinephrine (NE) in WKY and SHR rats.** NFκB activity assay showing increased activity in PVN of SHR + CP rats when compared to WKY + CP rats, whereas, VIPER infusion in SHR rats resulted in significant reduction in the NFκB activity. Similar trends were observed with regard to plasma norepinephrine levels as well. Values are means ± SE. n = 6/group for NFκB activity; n = 8 for plasma NE analysis. ***P <0.001. CP, control peptide; NE, norepinephrine; NFĸB, nuclear factor-kappa B; PVN, paraventricular nucleus; SHR, spontaneously hypertensive rat; TLR4, Toll-like receptor 4; TNF-α, tumor necrosis factor-alpha; VIPER, viral inhibitory peptide of TLR4; WKY, wistar-Kyoto.

We performed NFκB binding activity assay using the PVN tissues of all rat groups as illustrated in Figure 7A. As expected, we observed that SHR + CP rats had significantly higher NFκB activity in the PVN homogenates than WKY + CP rats. Interestingly, this response was abolished by chronic VIPER infusion within the PVN of SHR rats. However, VIPER infusion did not affect NFκB activity in the WKY rats.

### Toll-like receptor 4 blockade reduced circulating norepinephrine levels in hypertensive rats

SHR + CP group had an increased circulating plasma NE (Figure 7B) concentration when compared to WKY + CP rats. Interestingly, inhibition of TLR4 using VIPER in the PVN caused a significant reduction in the plasma levels of NE in SHR but not in WKY.

## Discussion

**Figure 8:**
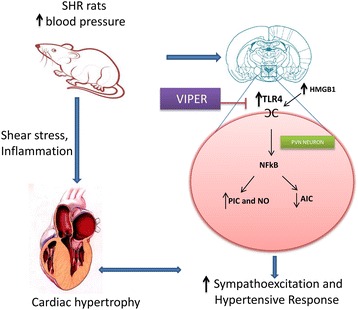
**Schematic illustrating proposed mechanism of TLR4 activation and downstream signaling in the PVN hypertension.** One of the mechanisms of sustained elevation of blood pressure in essential hypertension could be due to increased activation of TLR4 in the PVN and subsequent activation of NFκB pathway to produce inflammatory alterations, sympathoexcitation, and cardiac hypertrophy. Blockade of TLR4 in the PVN attenuates hypertensive response and prevents detrimental inflammatory changes associated with hypertension. Besides reduction in MAP in VIPER treated SHR rats, we also observed reduction in plasma NE levels. Therefore these beneficial effects might be also due to reduction in plasma NE and HMGB1 levels as observed in this study: HMGB1, high mobility group box 1; MAP, mean arterial pressure; NE, norepinephrine; NFĸB, nuclear factor-kappa B; PVN, paraventricular nucleus; SHR, spontaneously hypertensive rat; TLR4, Toll-like receptor 4; VIPER, viral inhibitory peptide of TLR4.

In the present study, we investigated the effects of bilateral inhibition of TLR4 within the hypothalamic PVN of the brain of SHR rats, which are well established as a genetic model of human essential hypertension. The salient findings of this study are as follows:

1. SHR rats had robust increase in TLR4 levels in the PVN, which was mostly localized in the neurons and microglia. These results were associated with increase in HMGB1 levels within the PVN as well as in the circulation.
2. Blockade of TLR4 within the PVN prevented, at least in part, the increase in blood pressure in SHR and attenuated cardiac hypertrophy in SHR.
3. TLR4 inhibition in the PVN attenuates pro-inflammatory cytokines, iNOS, and transcription factor, NFκB activity; whereas, TLR4 inhibition causes an increase in anti-inflammatory IL-10 in the PVN of hypertensive SHR rats.
4. TLR4 blockade resulted in significant reduction in circulating plasma NE in SHR rats. In the present study, we observed a significant increase in the TLR4 gene and protein expression within the PVN of hypertensive rats. More importantly, our double-labeling immunofluorescence staining of the frozen floating PVN sections demonstrated that TLR4 protein is present mainly in neurons and microglia (albeit at much lower level) and not so much in the astrocytes, indicating that TLR4 upregulation in neurons and microglia of the PVN could be one of the characteristics of hypertensive response observed in these animals. To the best of our knowledge, the present study is the first to demonstrate that increased TLR4 expression in the PVN contributes to a hypertensive response in a genetic model of hypertension. Furthermore, a corresponding increase in the HMGB1 levels in the PVN and in circulation indicates that TLR4 acts via the HMGB1. These results provide mechanistic evidence that detrimental effects seen in genetic hypertension are mediated, at least in part, by TLR4 in the PVN and that inhibition of TLR4 attenuates hypertensive response, possibly via downregulation of inflammatory components and upregulation of anti-inflammatory mediators in the PVN. Taken together, the results of this study suggest TLR4 as a newer therapeutic target for effective control of blood pressure. The proposed mechanism by which TLR4 exerts its role in hypertension is depicted in Figure 8.

Hypertension is a chronic inflammatory condition. In this context, immune system works as a first line of defense against an insult or tissue injury and acts via mechanism of inflammation. This critical response is initiated by identification of pathogen-associated molecular patterns by pathogen recognition receptors present on cell membrane [12]. TLRs are one such pathogen recognition receptors, which are considered a major component of the innate immune system. To date, 13 TLRs have been cloned in mammals and each TLR have a distinct function in innate immune recognition [35]. TLRs are type I transmembrane glycoproteins. They can be classified into two groups according to their subcellular localization: TLR1, TLR2, TLR4-6 and TLR11 are expressed on the plasma membrane, whereas TLR3, TLR7 and TLR9 are found in the endosomal compartment [36]. Human TLR4 was the first characterized mammalian TLR [37]. In addition to immune cells, TLR4 has also been found to be expressed in nonimmune cells of the cardiovascular system [38], brain [39,40], neuronal culture stimulated with LPS [12], and vascular smooth muscle cells [41], indicating that body tissues have control over their own immune response. Our current results together with previous reports suggest role of TLR4 in hypertension. Although hypertension is a chronic inflammatory condition, the role played by TLR4 , which responds to endogenous DAMPs, in pathogenesis of hypertension is largely unclear. Moreover, it is well known that the PVN is central in initiation and development of hypertension, the role of TLR4 within the PVN of genetic model of human essential hypertension has never been investigated before.

In this study, to test our hypothesis that hypertensive response observed in SHR rats could be mediated by activation of TLR4 in the PVN, we chronically infused a specific TLR4 blocker (VIPER) or control peptide (CP) by intra-PVN route in the SHR rats and their normotensive control, the WKY rats. We observed a significant reduction in MAP in the hypertensive rats that received VIPER (SHR + VIPER) when compared to the hypertensive rats receiving the control peptide (SHR + CP), and saw no comparable changes in WKY rats receiving VIPER. Additionally, our telemetric recordings showed a significant reduction in MAP in the SHR + VIPER group starting from day 4 of the VIPER infusion, which remained lower until the end of the experiment. In keeping with our results, a previous report showed that a neutralizing TLR4 antibody reduces blood pressure in the SHR rats [3]. The present study provided further evidence that inhibition of TLR4 in the PVN partially restored blood pressure to normal levels, supporting that TLR4, along with other central mechanisms, plays an important role in BP regulation [42-44].

Hypertension is characterized by increased cardiac hypertrophy and cardiac dysfunction. [45]. Therefore, we investigated whether TLR4 blockade in the PVN is cardioprotective in hypertensive rats. SHR + CP rats had increased ANP levels and HW:BW when compared with WKY + CP rats, indicating presence of cardiac hypertrophy in SHR as also reported in previous studies [30,46]. More importantly, TLR4 blockade within the PVN resulted in a significant reduction in ANP and the HW:BW ratio in hypertensive rats, suggesting reduced cardiac hypertrophy by TLR4 blockade. A handful of previous studies have reported that TLR4 deficiency protects the myocardium in Angiotensin II-induced hypertension in rat [40] and from ischemia/reperfusion injury in mice [47]. Taken together, the current findings suggest the role of brain TLR4 in cardiac hypertrophy in hypertension.

Each TLR recognizes specific pathogen-associated molecular patterns (PAMPs) in microbial injury. TLRs, especially TLR4, can also be stimulated by host-derived molecules, known as damage-associated molecular pattern molecules (DAMPs) [16,17]. Of several DAMPs, high mobility group box-1 (HMGB1) is the most important DAMP that has been implicated in various inflammatory conditions. In response to injury or infection, HMGB1 is released actively by immune cells and passively by insulted cells into the extracellular space and initiates an inflammatory response [15-17]. Therefore, one possible mechanism by which TLR4 is activated in hypertension could be via upregulation of HMGB1. Reinforcing this hypothesis, in the present study, hypertensive rats were found to have significantly increased levels of HMGB1 in the PVN as well as in circulation. Similar secretion of HMGB1 and expression of TLR4 was observed in ischemic brain injury [48]. However, the novel finding of the present study is that inhibition of TLR4 within the PVN resulted in a significant decrease in HMGB1 levels in the PVN and circulation. Given that the TLR4 blockade reduces MAP, it can be postulated that TLR4 plays role in hypertension, possibly via HMGB1. Whether other known ligands of TLR4, such as heat shock proteins, have any roles within the PVN of hypertensive animals is not clear at this time and needs to be investigated in detail. Nevertheless, the present findings provide strong evidence that TLR4 within the PVN plays a critical role in the pathogenesis of hypertension, at least in part, due to increased binding with its specific DAMP, HMGB1.

Once a TLR recognizes an endogenous DAMP, activation of inflammatory signaling begins [16,17]. Moreover, an inflammatory response in hypertension is characterized by a peripheral and central increase in various PICs, in particular, TNF-α and IL-1β [20,49,50]. In addition, PICs are known to induce iNOS expression in hypertension [22]. Therefore, to investigate whether TLR4 signaling within the PVN contributes to inflammatory response seen in hypertension, we measured the gene and protein expression of TNF-α, IL-1β and iNOS in the PVN tissue. We found that chronic TLR4 inhibition in hypertensive rats causes a significant reduction in TNF-α and IL-1β levels, as well as iNOS levels, in the PVN. Additionally, anti-inflammatory cytokine IL-10 was found to be significantly increased in VIPER-infused hypertensive rats. These results clearly suggest the role of TLR4, specifically within the PVN in inflammatory response in hypertension. Although not within the PVN, a handful of previous reports showed that systemic injection of anti-TLR4 antibody decreases serum levels of IL-6 in SHR [3] and systemically infused TLR4 antagonist reduces serum levels of inflammatory markers in heart failure animals [51]. Similarly, another study has shown that the TLR4 and PICs were increased in the heart tissues of the SHR rats [45]. It is noteworthy that although these studies show suppression of inflammation in the end-organs, none of the studies were able to point out the role of the brain in hypertension Given our current findings that TLR4 is dramatically upregulated within the PVN and that the TLR4 blockade attenuates MAP, our results suggest a vital role played by hypothalamic TLR4 in hypertensive response. Hence, an improvement in MAP and cardiac function by central inhibition of TLR4, as observed in the present study, could be attributable to a reduction in iNOS and PICs. Supporting our results, a recent study has demonstrated that ablation of iNOS delays cardiac hypertrophy and contractile dysfunction in mice with aortic banding-induced hypertension [52]. Binding of free HMGB1 to TLRs leads to the production and release of PICs [53]. Our findings that HMGB1 levels were increased both in the circulation and in the PVN together with increase in PICs ,iNOS and plasma NE suggest that TLR4 participates in the inflammatory process associated with hypertension leading to sustained activation of NFκB pathway to produce inflammatory alterations, sympathoexcitation, and cardiac hypertrophy in SHR rats.

NFκB is one of the most important downstream transcription factors responsible for the transcription of PICs and iNOS. It is also well established that TLR4 shares the same NFκB signaling pathways. Given that VIPER injection into the PVN of hypertensive rats reduces PICs and iNOS levels, one possible mechanism by which TLR4 inhibition in PVN exerts its beneficial effects could be via downregulation of NFκB. Reinforcing this hypothesis, the present study showed that the TLR4 blockade causes downregulation of NFκB activity in SHR rats. To the best of our knowledge, the present study is the first to investigate the downstream signaling mechanism of TLR4 within the PVN in regulation of MAP. Nevertheless, these results clearly suggest that attenuation of NFκB activity might be attributable to reduced inflammation, which in turn leads to disruption of a detrimental positive feedback cycle involved in cardiac remodeling and the progression of hypertension.

It has been well established that sympathetic hyperactivity contributes to cardiac remodeling in hypertension via an increase in TNF-α production [54] and NFκB activation leading to end organ damage [55]. In accordance with these previous reports, in the present study, the SHR rats showed higher circulating plasma levels of NE (an indirect indicator of sympathetic activity), indicating increased sympathetic outflow compared to WKY rats. However, a novel finding of this study is that inhibition of TLR4 in the PVN with VIPER resulted in a dramatic reduction in plasma levels of NE in the hypertensive rats, suggesting that upregulation of brain TLR4 plays a key role in sympathoexcitation as observed in hypertensive animals. The mechanism by which TLR4 increases sympathetic output in SHR is not clear at this time. However, given that inhibition of TNF-α or NFκB in the PVN decreases arterial pressure [30] and sympathetic activity [56], it is plausible to suggest that activation of TLR4 signaling induces sympathetic hyperactivity possibly via increased PICs. As evidence, Ogawa *et al*. demonstrated that siRNA-mediated inhibition of brain TLR4 decreases urinary norepinephrine excretion in rats with myocardial infarction [44]. Nonetheless, the results of the present study suggest that activation of TLR4 in the PVN of the brain leads to increased sympathetic activation in an animal model of essential hypertension.

### Limitations

There are two common pathways shared by TLRs, namely the myeloid differentiation factor 88 (MyD88)-dependent and the MyD88-independent pathways [57,58]. An increase in PICs expression induced through NFκB activation is the main feature of the MyD88-dependent pathway, whereas activation of interferon-γ regulatory factor 3 is characteristic of the MyD88-independent pathway [59,60]. Given our findings that the TLR4 blockade attenuates PICs and NFκB, we believe that the MyD88-dependent pathway is involved in this process. Specific adaptors and possible ligands, other than HMGB1 involved in TLR4 signaling, need to be investigated and could be a focus for future studies. Furthermore, here we explored the PVN region, although there are multiple cardiorelevant sites in the brain that can play a role in modulating the hypertensive response. However, we feel that the PVN is of importance because of its recognized integrative functions. Nonetheless, the current data undermines the key role played by brain TLR4 signaling in the pathogenesis of hypertension and suggest that TLR4 as a novel therapeutic target for the treatment of essential hypertension.

## Conclusions

To summarize, TLR4 regulates various diseases in an NFκB-dependent manner. It has been shown to increase cardiac hypertrophy and dysfunction in animal models of heart failure and hypertension. Here, we show that the SHR rats exhibit dramatic upregulation of TLR4 within the cellular components of the hypothalamic PVN. More importantly, inhibition of TLR4 by bilateral microinjection into the PVN of the SHR rats delays the progression of hypertension; reduces cardiac hypertrophy; attenuates PICs, iNOS, and NFκB, as well as NE levels; and improves anti-inflammatory IL-10 levels within the PVN. Additionally, our results identify TLR4 as a specific TLR, and HMGB1 as one of its endogenous ligands that plays a vital role in the hypertensive response. Taken together, the present study shows activation of HMGB1 as an upstream phenomenon and activation of inflammatory signals as a downstream pathway in TLR4 signaling within the PVN of hypertensive rats. Overall, our data for the first time demonstrate that an excessive increase in TLR4 within the PVN plays an important role in initiation of the inflammatory process observed in essential hypertension.

## Abbreviations

aCSF: Artificial cerebrospinal fluid
ANOVA: Analysis of variance
ANP: Atrial natriuretic peptide
BW: Body weight
*CD11B*: Cluster of differentiation molecule 11B
CP: Control peptide
ELISA: Enzyme-linked immunosorbent assay
GAPDH: Glyceraldehyde 3-phosphate dehydrogenase
GFAP: Glial fibrillary acidic protein
HMGB1: High mobility group box 1
HW: Heart weight
HW/BW: Heart weight/body weight
IACUC: Institutional Animal Care and Use Committee
IL-1β: Interleukin-1 beta
IL-10: Interleukin-10
iNOS: Inducible nitric oxide
MAP: Mean arterial pressure
MyD88: Myeloid differentiation factor 88
mmHg: Millimeters of mercury
NE: Norepinephrine
NeuN: Neuronal nuclei
NFĸB: Nuclear factor-kappa B
NO: Nitric oxide
PIC: Proinflammatory cytokine
PVN: Paraventricular nucleus
SHR: Spontaneously hypertensive rat
TLR: Toll-like receptor
TNF-α: Tumor necrosis factor-alpha
VIPER: Viral inhibitory peptide of TLR4
WKY: Wistar-Kyoto
